# Oncogenic Ras and ΔNp63α cooperate to recruit immunosuppressive polymorphonuclear myeloid-derived suppressor cells in a mouse model of squamous cancer pathogenesis

**DOI:** 10.3389/fimmu.2023.1200970

**Published:** 2023-08-10

**Authors:** Nozomi Sakakibara, Paúl E. Clavijo, Cem Sievers, Veronica C. Gray, Kathryn E. King, Andrea L. George, Roshini M. Ponnamperuma, Beatriz A. Walter, Zhong Chen, Carter Van Waes, Clint T. Allen, Wendy C. Weinberg

**Affiliations:** ^1^ Office of Biotechnology Products, Center for Drug Evaluation and Research, FDA, Silver Spring, MD, United States; ^2^ Translational Tumor Immunology, National Institute on Deafness and Other Communication Disorders, NIH, Bethesda, MD, United States; ^3^ Genitourinary Malignancies Branch, Center for Cancer Research, NCI, NIH, Bethesda, Maryland, MD, United States; ^4^ Head and Neck Surgery Branch, National Institute on Deafness and Other Communication Disorders, NIH, Bethesda, MD, United States

**Keywords:** PMN-MDSC, p63, ras, carcinogenesis, oncogenic, *in vivo*, tumor micro environment (TME), squamous

## Abstract

**Introduction:**

Amplification of human chromosome 3q26-29, which encodes oncoprotein ΔNp63 among other isoforms of the p63 family, is a feature common to squamous cell carcinomas (SCCs) of multiple tissue origins. Along with overexpression of ΔNp63, activation of the protooncogene, *RAS*, whether by overexpression or oncogenic mutation, is frequently observed in many cancers. In this study, analysis of transcriptome data from The Cancer Genome Atlas (TCGA) demonstrated that expression of *TP63 mRNA*, particularly *ΔNp63* isoforms, and *HRAS* are significantly elevated in advanced squamous cell carcinomas of the head and neck (HNSCCs), suggesting pathological significance. However, how co-overexpressed ΔNp63 and HRAS affect the immunosuppressive tumor microenvironment (TME) is incompletely understood.

**Methods:**

Here, we established and characterized an immune competent mouse model using primary keratinocytes with retroviral-mediated overexpression of ΔNp63α and constitutively activated HRAS (v-ras^Ha^ G12R) to evaluate the role of these oncogenes in the immune TME.

**Results:**

In this model, orthotopic grafting of wildtype syngeneic keratinocytes expressing both v-ras^Ha^ and elevated levels of ΔNp63α consistently yield carcinomas in syngeneic hosts, while cells expressing v-ras^Ha^ alone yield predominantly papillomas. We found that polymorphonuclear (PMN) myeloid cells, experimentally validated to be immunosuppressive and thus representing myeloid-derived suppressor cells (PMN-MDSCs), were significantly recruited into the TME of carcinomas arising early following orthotopic grafting of ΔNp63α/v-ras^Ha^-expressing keratinocytes. ΔNp63α/v-ras^Ha^-driven carcinomas expressed higher levels of chemokines implicated in recruitment of MDSCs compared to v-ras^Ha^-initiated tumors, providing a heretofore undescribed link between ΔNp63α/HRAS-driven carcinomas and the development of an immunosuppressive TME.

**Conclusion:**

These results support the utilization of a genetic carcinogenesis model harboring specific genomic drivers of malignancy to study mechanisms underlying the development of local immunosuppression.

## 1 Introduction

Human squamous cell carcinomas (SCCs) are derived from epithelial cells and share features across originating sites including head and neck, lung, esophagus, cervix and skin (1, 2). Vast datasets such as those available from The Cancer Genome Atlas (TCGA) Research Network have enabled bioinformatic analyses of cancers arising in these different tissue types (https://www.cancer.gov/tcga (3, 4)). Genomic and transcriptional analyses by Pan-TCGA revealed that chromosome 3q gain is a common molecular signature across all SCCs, estimated at up to 69% (2, 5, 6). At the heart of the amplified region of 3q26-3q29, *TP63*, a master transcriptional regulator of epithelial cell fate, is expressed predominantly as the ΔNp63 isoform in SCCs (2, 5, 6). The ΔNp63 isoform has been shown to play major roles in the establishment and maintenance of epithelial cell lineage, proliferation, and adhesion as well as the inhibition of differentiation and senescence. These activities are dependent on its expression level in a context-dependent manner (7–9). When overexpressed as in SCCs, the ΔNp63 isoform, lacking the N-terminal transactivation domain of p63, has a dominant-negative effect on p53 function and regulated genes, while promoting transactivation of a distinct gene repertoire through interaction with other transcription factors (9–11).

We previously established a murine genetic SCC tumor progression model utilizing primary epidermal keratinocytes that are transduced with retrovirus encoding Harvey rat sarcoma virus oncogene, v-ras^Ha^, with activating mutation at G12R alone or in the presence of lentiviral-driven ΔNp63α, and orthotopically grafted onto athymic nude mice hosts (12). We observed that the overexpression of ΔNp63α in combination with oncogenic v-ras^Ha^ enhances malignant conversion, in contrast to the development of papillomas observed with Ras alone (12). The role of oncogenic v-ras^Ha^ in neoplastic transformation has been attributed to activation of downstream effectors of receptor tyrosine kinases, which establishes a pro-inflammatory environment (13, 14). The cooperation of v-ras^Ha^ and ΔNp63α in malignant conversion can be explained, in part, by the role of ΔNp63α in overcoming v-ras^Ha^-induced senescence by inhibition of p16^ink4a^ and p19^arf^ expression (12, 15, 16). In addition to the anti-senescent role of ΔNp63α in driving malignancy, mounting evidence supports a role of ΔNp63α in orchestrating inflammation mediated by its interactions with NF-κB subunits (reviewed in (17)). We previously identified that the overexpression of ΔNp63α induces nuclear localization and activation of the NF-κB subunit, c-Rel, and regulates inflammatory response genes in primary murine keratinocytes [(18); King and Weinberg, unpublished results]. Furthermore, in human head and neck squamous cell carcinoma (HNSCC) cell lines, cREL and ΔNp63 form a complex in response to the inflammatory cytokine TNFα to activate NF-κB and AP-1 pathways (19–21). In addition, transgenic (TG) mouse models of overexpressed ΔNp63α in epidermis display hyperplasia, infiltration of immune and inflammatory cell populations (21–23), and enhanced malignant progression of chemically-induced tumors (24). The immune and inflammatory cells identified in ΔNp63α-overexpressing hyperproliferative epidermis included CD3^+^ T cells, CD4^+^ T cells, CD4^+^/CD25^+^/Foxp3^+^ regulatory T cells (Tregs), and M2 type macrophages, indicating that cell subsets implicated in both pro-inflammatory and immunosuppressive functions are recruited by prolonged overexpression of ΔNp63α in the epidermis. This appears to be mediated by increased levels of pro-inflammatory cytokines regulated by NF-κB (22). The activated NF-κB signaling and ΔNp63 expression levels showed positive correlation in HNSCCs, which are also enriched in immune components based on genomic analyses (2, 5, 6).

Considering the prevalence of human SCCs with elevated levels of ΔNp63 and increased immune infiltrates, and the co-activation of NK-κB/c-Rel with ΔNp63α, we investigated how ΔNp63α impacts the tumor microenvironment (TME) and its relationship to carcinoma formation. We adapted the orthograft model to evaluate the contributions of v-ras^Ha^/ΔNp63α in the athymic mouse background described above to immune-competent syngeneic hosts to characterize the complete composition of immune infiltrates in the v-ras^Ha^ or v-ras^Ha^/ΔNp63α-induced TME. Our data suggest that ΔNp63α and oncogenic v-ras^Ha^ cooperate to establish an immunosuppressive TME that promotes carcinogenesis.

## 2 Materials and methods

### 2.1 Animals

All animal work was performed in accordance with established NIH (National Institutes of Health) guidelines, following accepted standards of humane animal care under protocols approved by the Animal Care and Use Committee of the Center for Biologics Evaluation and Research of the Food and Drug Administration. Wild-type BALB/cAnNCr mice (BALB/c; strain code: 555) used to both establish syngeneic donor cell cultures and as grafting hosts were obtained from Charles River Laboratories, Kingston, NY.

### 2.2 Cell culture

Primary keratinocytes and fibroblasts were isolated and cultured from BALB/c newborn pups less than 4 days old, as previously described (25, 26). Keratinocytes were cultured in EMEM (Lonza, Catalogue #: 06-174G) with 8% chelexed fetal bovine serum (FBS) (Gemini, Catalogue #: 100-106) at a final calcium concentration of 0.05 mM (low calcium EMEM). Fibroblasts were cultured for 8-9 days in DMEM (Lonza, Catalogue #: 12-733F) with 10% newborn calf serum (NBCS) (Gibco, Catalogue #: 16010-159), prior to use in grafting studies.

### 2.3 Viruses and retroviral transduction of primary keratinocytes

A Ψ^2^ retrovirus packaging cell line was used to introduce the Ha-MSV gene from Harvey murine sarcoma virus (single G12R mutation; v-ras^Ha^) as previously described (27, 28). Lentivirus construct encoding human ΔNp63α (LV-ΔNp63α) under the FerH promoter was described previously (12). The empty vector construct, also referred to as Stuffer control, contains the FerH promoter followed by multiple stop codons (with no start codons) and is thus unable to initiate transcription driven by the FerH promoter. The construct was purchased from Protein Expression Laboratory, Leidos Biomedical Research Inc., Frederick National Laboratory for Cancer Research (Construct name: 17506-M36-685). Lentiviruses were generated from the constructs and titered by Cellomics Technology, LLC. Three days post-plating (a day after the transduction of retrovirus encoding v-ras^Ha^), the primary keratinocytes were incubated in fresh low calcium EMEM with lentivirus at 3x10^6^ Titer Unit (TU) per 60 mm^2^ dish (=1.4x10^5^ TU/cm^2^, a total number of cells estimated to be 1-2 x10^6^ cells), and 4 μg/ml of polybrene with MOI of 1.5-3 (final volume of 0.5 ml/60 mm^2^ dish), for 3 hours at 37˚C with rocking every 20 minutes. Fresh medium was added at the end of the incubation to bring the total volume to 3.5 ml/60 mm^2^.

### 2.4 Grafting

Primary murine newborn epidermal keratinocytes were transduced with v-ras^Ha^ and ΔNp63α or Stuffer as described above. After 9 days in culture (6 days post-transduction of keratinocytes with ΔNp63α), the keratinocytes and fibroblasts were trypsinized, collected, counted and aliquoted for grafting at 4x10^6^ keratinocytes and 8x10^6^ fibroblasts per mouse. The cells were deposited on the subcutaneous surface inside silicone domes that were implanted onto the mid-dorsum of the host (6-12 weeks old), as previously described (12). Both donor cells and hosts were of the wild-type BALB/cAnNCr strain. The domes were removed one-week post-surgery, and the tumors and grafted sites were collected at the time points indicated.

### 2.5 Antibodies

The following primary antibodies and conditions were used for western blotting: 1) p63, BiocareMedical anti-p63 antibody clone 4A4, 1:500 in 5% milk overnight at 4˚C. This antibody is directed against amino acids 1-205 of ΔNp63 and the epitope has been mapped to sequences that are shared with p73 (29, 30); 2) Ras, Sigma-Aldrich anti-Ras antibody clone RAS10, 1:2,000 in 5% milk overnight at 4˚C; 3) Beta-actin, Cell Signaling Technology clone 8H10D10, #3700, 1:10,000 in 5% milk overnight at 4 ˚C. The blots were incubated with secondary antibodies and imaged using ECL reagent available from Kindle Biosciences, LLC (Catalogue # R1004) following the product manual. The blots were imaged using KwikQuant Imager (Kindle Biosciences, LLC, Catalogue # D1001).

### 2.6 Flow cytometry

Single cell suspensions generated *via* mechanical dissociation of spleen or digestion of tumor with the Mouse Tumor Dissociation Kit (Miltenyi) as per manufacturer recommendations were incubated with CD16/32 (FcR block) antibodies for 10 minutes. Cells were stained with primary antibodies for 30 minutes. Anti-mouse CD45.2 (clone 104), CD11b (M1/70), Ly-6C (HK1.4), Ly-6G (1A8), CD3 (145-2C11), CD8 (53–6–7), CD4 (GK1.5), CD25 (PC61.5.3), FoxP3 (FJK-16s), and NK1.1 (PK136) antibodies were purchased from Biolegend or eBioscience. 7AAD was used to determine cell viability and a “fluorescence minus one” method was used to determine antibody specificity. For intranuclear staining, cells were fixed and permeabilized using a FoxP3 staining kit (eBioscience) per manufacturer’s protocol. All samples were analyzed on a BD FACSCanto analyzer using FACSDiva software. Post-acquisition analysis was performed using FlowJo vX10.0.7r2.

### 2.7 Multiplex immunofluorescence staining and multispectral analysis

Formalin-fixed paraffin-embedded sections (5 μm) were stained according to previously described methods (31). Antibodies used (target, clone or catalogue number, and sourcing) were as follows: mouse Ras (clone 18/Ras), BD Bioscience; ΔNp63 (Poly6190), Biolegend; CD4 (4SM95), CD8 (4SM15), and FoxP3 (FJK-16s), eBioscience; and Ly-6G (1A8), BD Pharmingen. Multispectral images were acquired using a Polaris system (Perkin Elmer/Akoya). Scanned images were digitized and individual cell types were quantified using inForm digital pathology analysis software (Akoya) per manufacturer recommendations.

### 2.8 Genomics analysis

The canSAR Black database was used to compare HRAS and TP63 isoform expression in different cancer types (32). Normalized isoform and gene expression data from The Cancer Genome Atlas (TCGA) were downloaded from firebrowse (http://firebrowse.org/), analyzed in R, and processed and visualized using Tidyverse (https://cran.r-project.org/web/packages/tidyverse/citation.html). Published single-cell RNA-seq (scRNA-seq) data were obtained from Puram et al. (33); processed expression data were downloaded from Gene Expression Omnibus (GSE103322) and subjected to log2 transformation after adding one to each value. Statistical analysis was perfomed in ggpubr (https://CRAN.R-project.org/package=ggpubr). From this single-cell RNA-seq dataset, only tumors yielding 50 or more tumor cells were considered for analysis (10 tumors).

### 2.9 Cytokine expression by qPCR

A Custom RT^2^ profiler PCR array (Qiagen) was used to profile mRNA expression of chemokines and their receptors in RNA samples isolated from tumors and from cultured primary keratinocytes. The assays were performed and analyzed according to the manufacturer’s instructions.

### 2.10 *In vitro* chemokine and cytokine protein expression by dot blot array

Primary keratinocytes were sequentially transduced with viral vectors encoding v-ras^Ha^, and either ΔNp63α or Stuffer, as described above. Three days post-transduction of lentivirus-ΔNp63 or Stuffer, the cell culture medium was replaced with fresh medium; 24 hours later the culture supernatant was collected and immediately incubated with a dot blot antibody array at 4°C overnight (Mouse Cytokine Array C1000, Raybiotech) according to the manufacturer’s instructions. The image was developed using Amersham ImageQuant LAS 4000.

### 2.11 *In vitro* cytokine quantitative immunoassay

Primary keratinocytes were sequentially transduced with viral vectors encoding v-ras^Ha^, and ΔNp63α or Stuffer, as described above. At three days and 13 days post-transduction of lentivirus-ΔNp63 or Stuffer, the cell culture medium was replaced with fresh medium and 24 hours later the culture supernatant was collected after centrifugation and frozen at -80°C until the day of the ProcartaPlex assay. Three independent biological experiments were performed. Cytokine assays were performed using multiplex bead-based kits for the indicated mouse cytokines per the manufacturers’ instructions (ProcartaPlex Immunoassays, ThermoFisher Scientific, CA). A total of 4 cytokines were assessed: CXCL1, CXCL5, CCL2, CCL20. Fluorescence of beads was measured using a Luminex Bioplex 200 analyzer (Bio-Rad Laboratories, Hercules, CA, USA), and data analysis was performed using the BioPlex Manager software (BioHercules, CA. USA) based on a five-parametric logistic nonlinear regression curve-fitting algorithm.

### 2.12 T cell proliferation assay

A T lymphocyte proliferation assay was performed as previously described (34). CD4^+^ and CD8^+^ T cells were isolated from naïve B6 spleens using the Pan T-Cell Kit (Miltenyi Biotec, negative selection) on an autoMACS Pro Separator (Miltenyi Biotec), labeled with a fluorescence dye 5 (6)-Carboxyfluorescein diacetate N-succinimidyl ester (CFSE, Sigma), and stimulated with plate-bound anti-CD3 (clone 145-2C11, eBioscience) and -CD28 (Clone 37.51, eBioscience) antibodies. T cells were co-cultured at a 1:2 ratio with putative MDSCs isolated from spleens or harvested from tumors derived from v-ras^Ha^/Stuffer (= empty vector) or v-ras^Ha^/ΔNp63 expressing keratinocytes. Granulocytic myeloid cells were isolated from spleens using the Anti-Ly6G Microbead Kit (Miltenyi Biotec, positive selection). To enrich tumor-infiltrating granulocytic myeloid cells, a 40/80% isotonic Percoll (Sigma) gradient (centrifuged at 325 × *g* for 23 minutes at room temperature) was followed by positive selection using the anti-Ly6G Microbead Kit. Flow cytometry was used to quantify CFSE dilution at 72-hours. Proliferation was quantified as the average number of divisions of all cells in the culture (division index) using commercially available FlowJo software v10.8.2 (35).

### 2.13 Statistics

Test of significance between pairs of data are reported as p-values, derived using a student’s t-test with a two tailed distribution and calculated at 95% confidence. Comparison of multiple sets of data was achieved with analysis of variance (ANOVA) with Tukey’s multiple comparisons. All error bars indicate standard error. Statistical significance was set to p < 0.05. All analyses were performed using GraphPad Prism v7 unless otherwise indicated.

## 3 Results

### 3.1 Upregulation of *HRAS* and *TP63* expression in human squamous cell carcinoma

**Figure 1:**
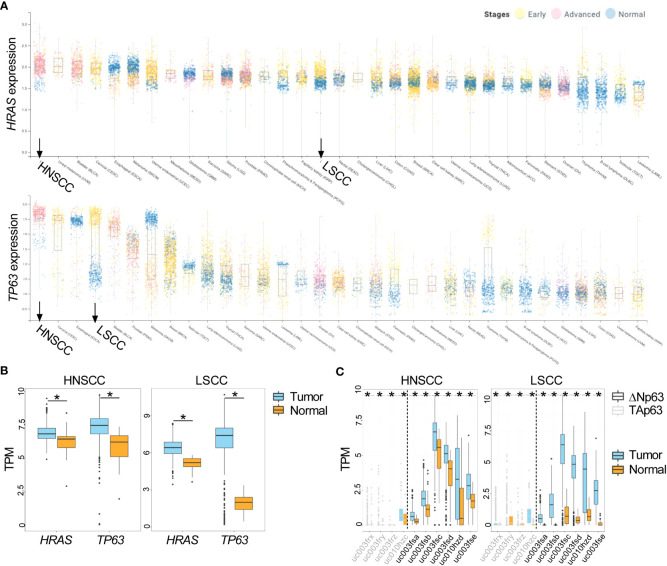
Increased expression of *HRAS* and *TP63* mRNA in TCGA data. **(A)** Expression of *HRAS* (upper panel) and *TP63* (lower panel) for stage I and II (early, yellow data points) or stage III and IV (advanced, pink data points) cancers along with corresponding normal tissue (blue data points) is shown as box and whisker plots. HNSCC and LSCC are highlighted in bold. **(B)** Box and whisker plots show gene expression of *HRAS* and *TP63* within tumor (in light blue) and normal (in gold) samples for HNSCC (left panel) and LSCC (right panel) obtained from TCGA. **(C)** Box and whisker plots show the expression of *ΔNp63* (black) and *TAp63* (grey) isoforms in tumor (in light blue) and normal (in gold) samples for HNSCC (left panel) and LSCC (right panel) obtained from TCGA. The x-axis labels represent TCGA transcript identifiers corresponding to *ΔNp63* and *TAp63* isoforms. HNSCC, head and neck squamous cell carcinoma; LSCC, lung squamous cell carcinoma. *, *p* < 0.05, Wilcoxon test.

Large databases allow for analysis of common pathways and oncogenes aberrantly expressed across diverse cancer types. Using the canSAR database (http://cansarblack.icr.ac.uk (36, 37)), which includes data from The Cancer Genome Atlas (TCGA), we analyzed *TP63* and *HRAS* gene expression in multiple cancer types. Study of the TCGA data corroborates that both *HRAS* and *TP63* expression are significantly elevated in advanced stage HNSCCs and early-stage lung squamous cell carcinoma (LSCC) compared to normal tissue (**Figures 1A, B**). The expression of each of these genes in HNSCCs ranked the highest among the major cancer types analyzed, suggesting oncological significance. We further demonstrate that ΔNp63 isoforms are expressed to a greater degree than TAp63 isoforms in these cancer types (**Figure 1C**), consistent with earlier reports (2, 5).

**Figure 2:**
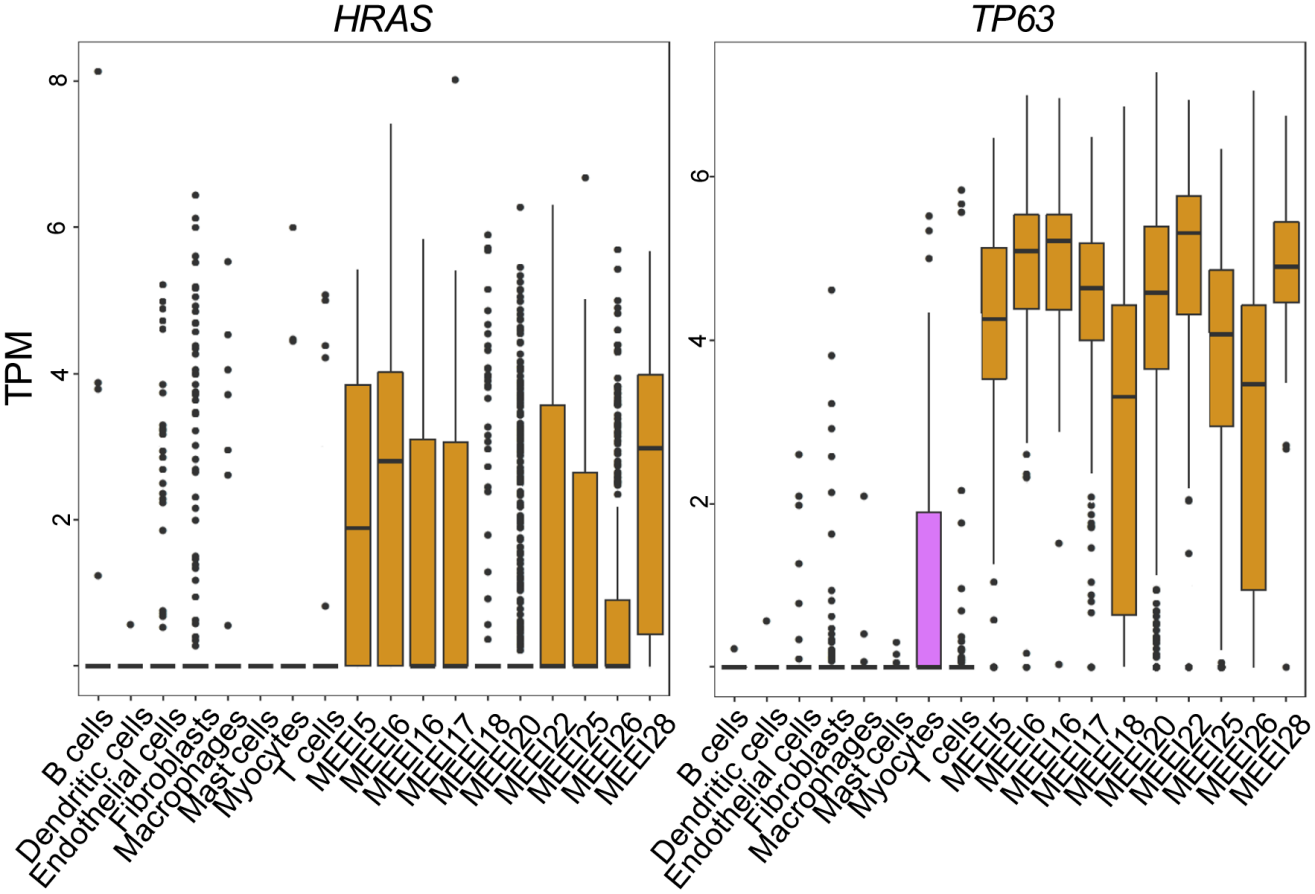
Single Cell RNA sequencing reveals increased expression of *HRAS* and *TP63* in malignant HNSCC cells. Box and whisker plots show mRNA levels of *HRAS* (left panel) and *TP63* (right panel) within malignant (prefixed MEEI) and non-malignant cells from HNSCC profiled by publicly available single-cell RNA sequencing (33). TPM, transcripts per million.

A limitation of the application of bulk genomic data from TCGA is the inability to distinguish the heterogeneity that exists in gene expression across different cell populations within the TME. To evaluate the expression of TP63 and HRAS within individual cell types, we utilized previously published scRNA-seq data generated from primary HNSCC tumors (33). Data presented in **Figure 2** indicate that *TP63* and *HRAS* expression is generally greater in malignant epithelial cells compared to non-malignant cell populations, such as immune cells and stromal cells. These data indicate that the increased expression of *TP63* and *HRAS* observed in bulk genomic data is likely due to increased expression in tumor cells, with limited contribution from immune or stromal cells.

### 3.2 Adaptation of the immune deficient orthotopic mouse model of SCC to a syngeneic immune competent host

**Figure 3:**
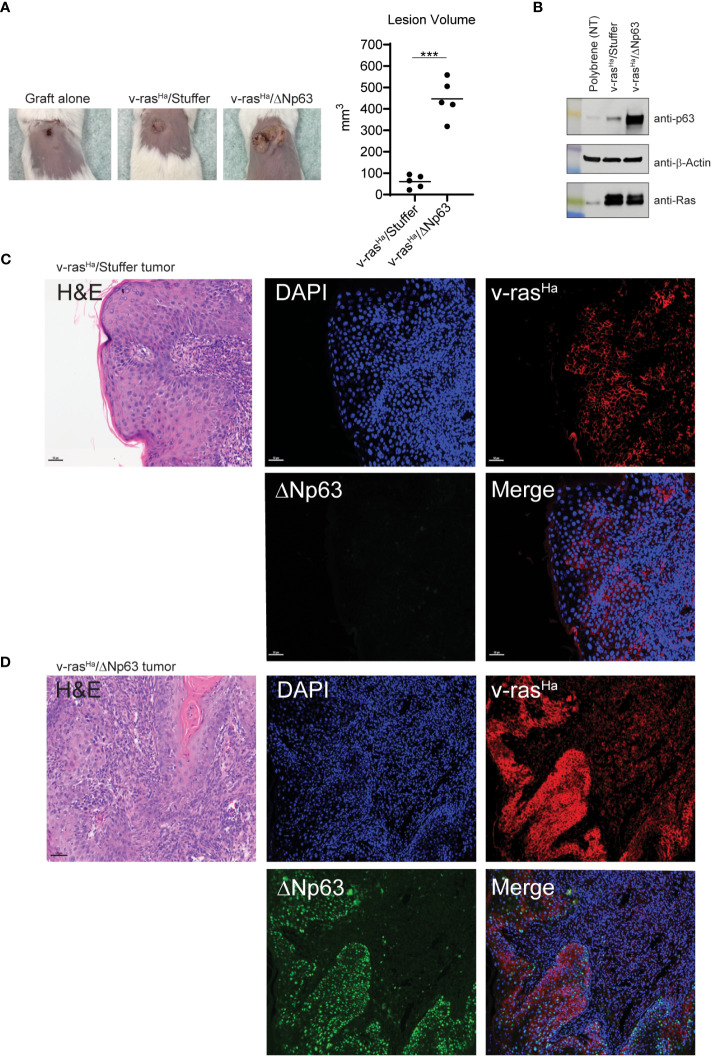
*In vivo* grafts derived from engineered keratinocytes retain expression of exogenous v-ras^Ha^ and ΔNp63. **(A)** Primary control keratinocytes (not virally transduced, incubated with polybrene alone) or primary keratinocytes transduced with viruses encoding *v-ras^Ha^* and *Stuffer* or *v-ras^Ha^* and *ΔNp63* were combined with cultured primary dermal cells and grafted onto the dorsal surfaces of syngeneic BALB/c mice. Representative images (10X lens) of graft sites 4 weeks post-grafting (5 animals per group) are shown on the left; quantification of lesion volume is shown on the right. No tumor growth was observed from the control grafts. **(B)** Control keratinocytes (polybrene exposure alone) or parallel cultures transduced with virus *v-ras^Ha^* alone or in combination with *ΔNp63* were assessed for protein expression by western blot analysis. **(C, D)** Primary keratinocytes transduced with viruses encoding v-*ras^Ha^* and *Stuffer* **(C)** or *v-ras^Ha^* in combination with *ΔNp63* **(D)** were grafted onto the backs of syngeneic BALB/c mice. After 4 weeks lesions were harvested, fixed, and stained for v-ras^Ha^ (red) or ΔNp63 (green) protein expression by immunofluorescence. DAPI (Blue) nuclear counterstain. ***, *p* < 0.001. Stuffer = Empty Vector.

We previously described an orthotopic murine graft model that uses primary epidermal keratinocytes transduced with retroviral vectors to overexpress oncogenic v-ras^Ha^ and wildtype ΔNp63 with immune deficient athymic nude mice as hosts (12), to evaluate the contribution of ΔNp63 and v-ras^Ha^ to squamous cancer pathogenesis. Overexpression of v-ras^Ha^ in this model mimics the RAS activation in human SCCs (**Figure 1**) by oncogenic mutation or overexpression of wild type gene. Likewise, lentiviral-driven ΔNp63 elevated expression of ΔNp63 mimics the gene amplification and overexpression of ΔNp63 observed in human SCCs. Mouse cutaneous SCC (cuSCC) models have been described to harbor molecular similarities and parallels not only to human cuSCCs but to SCCs arising from other tissues as well (38, 39). Our orthograft model reflects the genetic alterations observed in human cancers of head and neck and lung (**Figures 1**, **2**), and has served as a useful tool to decipher the implications of these genetic changes. Indeed, events associated with ΔNp63α overexpression that were identified in this cutaneous model, such as activation of NF-κB/c-Rel, have been confirmed in human HNSCC samples and cell lines (18). The observation that overexpression of ΔNp63α can induce an immune response in mice (21, 22) further suggested that this orthograft system could be adapted to explore the full complement of immune components modulated during v-ras^Ha^ -initiated tumorigenesis and ΔNp63α-dependent malignant conversion, as a model of human HNSCCs that frequently harbor amplified p63 and are often heavily infiltrated by inflammatory cells (40). We therefore adapted the athymic mouse model to an immunocompetent syngeneic background with BALB/c mice as hosts. As shown in **Figure 3**, orthotopic grafting of BALB/c primary epidermal keratinocytes that had been transduced with a retroviral vector encoding v-ras^Ha^ and a control lentiviral vector (Stuffer) along with primary dermal cells results in papilloma formation, while grafting of primary keratinocytes from BALB/c mice transduced with retroviral vectors encoding v-ras^Ha^ and ΔNp63α yields carcinomas, consistent with previous findings in athymic nude mice (12). No lesions were observed following grafting of control primary keratinocytes or keratinocytes overexpressing ΔNp63α alone (**Figure 3A**; **Supplementary Figure 1**), consistent with our previous findings (12). Western blot and immunofluorescence staining of the tissue sections confirm the expression of v-ras^Ha^ and ΔNp63α (**Figures 3B, C**). H&E staining confirms predominant papilloma vs carcinoma histology as early as 2 weeks post grafting in v-ras^Ha^- and v-ras^Ha^/ΔNp63α - expressing tumors respectively (**Supplementary Figure 2**).

### 3.3 The mRNA levels of chemokines and chemokine receptors known to mediate recruitment of immunosuppressive cells are elevated in v-ras^Ha^/ΔNp63α tumors

Chemokines are known to mediate immune cell trafficking in the tumor microenvironment (TME) and are secreted by both tumor and stromal cells. Our previous and on-going observations indicate that overexpression of ΔNp63α in primary murine keratinocytes promotes interactions with the c-Rel subunit of NF-κB and activates genes that are associated with inflammation (18), King and Weinberg, unpublished observations). In transgenic mice, elevated levels of ΔNp63α in the epidermis activate expression of pro-inflammatory chemokines that cooperate with NF-κB transcription factors to promote immunosuppressive type 2 chemokines and cytokines, consistent with the deregulated inflammatory response observed in human HNSCCs (21–23). To gain insight into whether these chemokines are differentially regulated in epithelial cell populations that give rise to benign versus malignant tumors, we used a commercially available cytokine array to examine the chemokines and cytokines produced *in vitro* by these keratinocyte populations. In this experiment, supernatants from cultured primary murine keratinocytes transduced with 1) empty vector alone (“Stuffer” control), 2) empty vector in combination with v-ras^Ha^ (v-ras^Ha^/Stuffer), 3) ΔNp63α alone, and 4) the combination of v-ras^Ha^ and ΔNp63α (v-ras^Ha^/ΔNp63α) were screened for 96 mouse cytokines and chemokines using multiplexed mouse cytokine antibody array (RayBiotech). A qualitative analysis revealed upregulation of 16 secreted factors by v-ras^Ha^/Stuffer- and v-ras^Ha^/ΔNp63α-transduced cells, and 2 downregulated proteins (**Supplementary Figures 3A, B**; **Table S1**). Specifically, increased levels of CXCL1, CXCL2, CXCL5, CXCL7, CXCL16, CCL2, CCL20, IGFBP-3, MMP-3, and OPN were observed in the supernatant of v-ras^Ha^/Stuffer- and v-ras^Ha^/ΔNp63α-transduced cells. Many of these chemokines and cytokines are known to play a role in chemotaxis of immune and immunosuppressive cells, including myeloid-derived suppressor cells (MDSC), tumor associated macrophages (TAM), monocytes, and neutrophils (41–46). Notably, there was no significant change in the cytokine profile between the control and ΔNp63α- transduced cells. Relative protein levels of CXCL1, CXCL5, CCL2, and CCL20 were further evaluated using the ProcartaPlex method, with similar findings (**Supplementary Figure 3C**). To rule out the possibility that the method may not be sufficiently sensitive to detect small changes by ΔNp63α alone, we evaluated the mRNA levels of MDSC- and Treg-recruiting chemokines *Cxcl1, Cxcl2, Cxcl5, Ccl1, Ccl17*, and *Ccl22* in keratinocytes expressing v-ras^Ha^ and ΔNp63α either separately or together using RT^2^-custom PCR arrays (**Supplementary Figure 4**). The data indicate that overexpression of v-ras^Ha^ upregulates *Cxcl1*, *Cxcl2*, and *Cxcl5*, while ΔNp63α downregulates the expression of these chemokines (**Supplementary Figure 4**). Taken together, these data suggest that the enhanced chemokine/cytokine production was mainly driven by v-ras^Ha^ expression in the *in vitro* setting.

**Figure 4:**
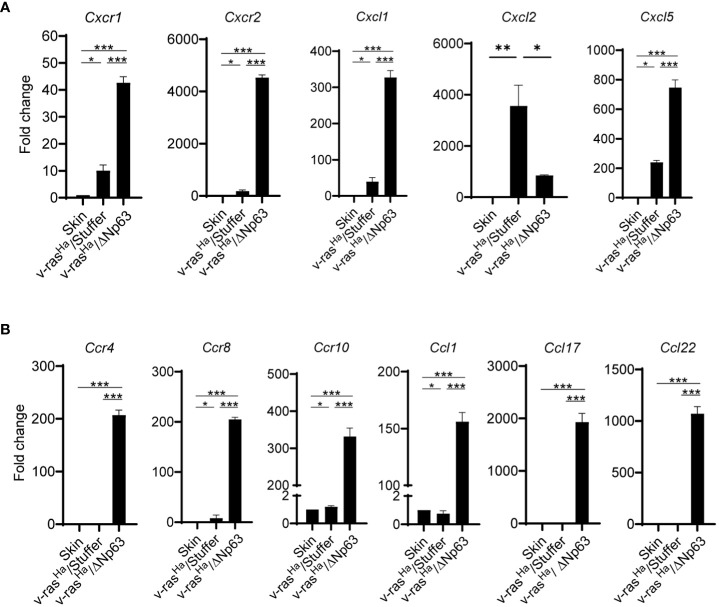
*In Vivo* chemokines and chemokine receptors are highly expressed in Ras/ΔNp63 carcinomas relative to Ras initiated papillomas. RNA isolated from skin or lesions derived from grafted primary keratinocytes encoding *v-ras^Ha^* and Stuffer control (v-ras^Ha^/Stuffer) or *v-ras^Ha^* in combination with *ΔNp63* (v-ras^Ha^/ΔNp63) at 2 weeks post grafting were analyzed for the expression of chemokines and chemokine receptors involved in the trafficking of myeloid cells **(A)** or Tregs **(B)** by qRT-PCR using a custom RT^2^ qPCR profiler. Three tumors per group were tested and analyzed. Quantified as fold change relative to normal skin. *, *p* < 0.05; **, *p* < 0.01; ***, *p* < 0.001. Stuffer = Empty Vector.

In light of this finding, we evaluated whether these chemokines are similarly deregulated *in vivo* in the murine tumor context, using the same RT^2^-custom PCR arrays. We specifically focused on the expression of genes involved in immunosuppression during early establishment of the tumor (2 weeks post-grafting). As shown in **Figure 4A**, the mRNA levels of chemokine receptors on MDSCs, *Cxcr1* and *Cxcr2*, as well as corresponding ligands, *Cxcl1* and *Cxcl5*, are significantly increased in the tumors derived from v-ras^Ha^/ΔNp63α expressing keratinocytes compared to tumors expressing v-ras^Ha^ in the absence of p63, or intact skin. In contrast, v-ras^Ha^-initiated papillomas upregulated the expression of the *Cxcr2* ligand *Cxcl2* in comparison to intact skin or Ras/ΔNp63α carcinomas. The *Cxcl1* and *Cxcl5* expression levels were also increased in v-ras^Ha^ tumors compared to control but to a lesser degree, with lower statistical significance compared to v-ras^Ha^/ΔNp63α tumors. Additionally, the mRNA levels of Treg chemokine receptors *Ccr4, Ccr8*, and *Ccr10* as well as their ligands, *Ccl1, Ccl17*, and *Ccl22* are significantly upregulated in the v-ras^Ha^/ΔNp63α carcinomas compared to v-ras^Ha^/Stuffer tumors or normal skin (**Figure 4B**). These data support that ΔNp63α cooperates with v-ras^Ha^ *in vivo* to promote the production of chemokines implicated in driving the recruitment of cells with Treg and MDSC markers and immunosuppressive function into the TME.

### 3.4 ΔNp63α expressing carcinomas have increased numbers of PMN-MDSCs recruited into the TME

To investigate whether these cytokine expression patterns correspond to distinct host immune profiles in papillomas relative to carcinomas, grafts of v-ras^Ha^- and v-ras^Ha^/ΔNp63α-expressing primary keratinocytes were harvested at 2, 3, and 4 weeks post-grafting and immune infiltration profiles were determined by flow cytometry. The tumors were screened for the presence of (CD11b^+^Ly6G^hi^Ly6C^int^) polymorphonuclear (PMN)-like myeloid cells, CD11b^+^Ly6G^lo^Ly6C^hi^ monocytic myeloid cells, CD8^+^ T-cells, CD4^+^ T-cells, and CD4^+^CD25^+^FOXP3^+^ regulatory T-cells (Tregs).

**Figure 5:**
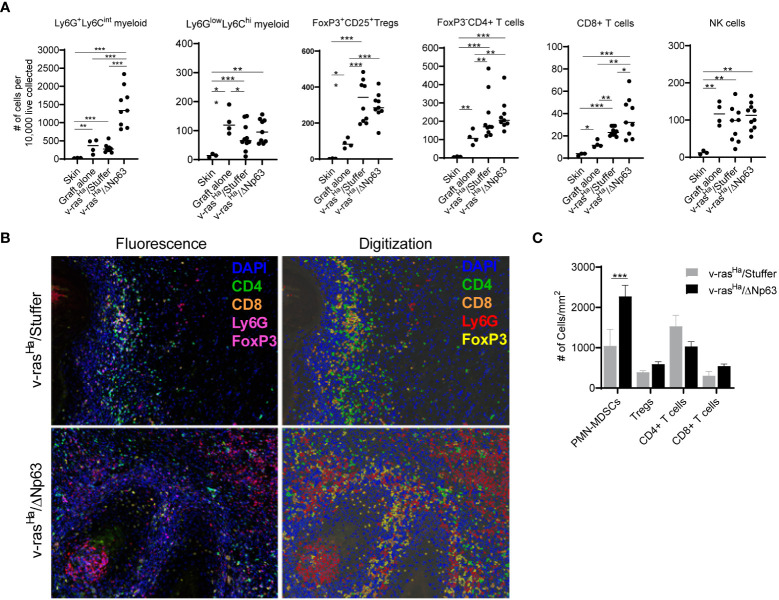
v-ras^Ha^ and ΔNp63 co-operatively drive tumor inflammation. **(A)** Intact skin, graft sites receiving control cultured primary keratinocytes (no virus transduced), or tumors derived from grafted primary keratinocytes encoding *v-ras^Ha^* + empty vector (v-ras^Ha^/Stuffer) or *v-ras^Ha^* in combination with *ΔNp63* (v-ras^Ha^/ΔNp63) were assessed for inflammatory cell infiltration by flow cytometric analysis. Cell count was normalized to number of cells per 1x10^4^ live cells collected to account for different sizes of lesions. All cells quantified are live, CD45^+^. Ly6G^hi^Ly6C^int^ (Ly6G^+^Ly6C^int^) and Ly6G^low^Ly6C^hi^ myeloid cells are CD11b^+^. Tregs are CD4^+^FoxP3^+^CD25^+^; CD4^+^ T cells are CD3^+^CD8^-^CD4^+^FoxP3^-^; CD8+ T cells are CD3^+^CD8^+^CD4^-^; NK cells are NK1.1^+^CD3^-^. Each dot represents a single lesion from a single mouse. All data from two independent experiments were pooled. **(B)** Multiplex immunofluorescence was used to assess tumor infiltration of inflammatory cells. Representative photomicrographs (10X lens) of lesions derived from primary keratinocytes encoding *v-ras^Ha^* alone (v-ras^Ha^/Stuffer, top panels) or in combination with *ΔNp63* (v-ras^Ha^/ΔNp63, bottom panels) are shown on the left, and representative digital images allowing for automated quantification are shown on the right. Note that FoxP3 and Ly6G are both stained with magenta. FoxP3+ (Tregs) cells have nuclear stain and Ly6G + cells have cytoplasmic stain and appear as rings. **(C)** Quantification of tumor infiltrating inflammatory cells from at least 5 high power fields (HPF) per lesion. Results shown in **(A, C)** are pooled from two independent assays each performed in multiple technical replicates. *, *p* < 0.05; **, *p* < 0.01; ***, *p* < 0.001. Stuffer = Empty Vector.

The immune landscape changed over a 4-week period during the development of the tumors, and there were notable differences in the immune profiles between tumors arising from grafts of v-ras^Ha^ -expressing keratinocytes compared to those expressing v-ras^Ha^/ΔNp63α at 2, 3, and 4- weeks post-grafting (data not shown). The most notable differences in immune cell components were seen at week 2 post-grafting, therefore the experiment was repeated with tumors harvested at this peak timepoint. CD4^+^ and CD8^+^ T-cells can exert effector function and regulate tumor growth and are typically associated with good prognosis (47). As shown in **Figure 5A**, both v-ras^Ha^ and v-ras^Ha^/ΔNp63α-induced tumors recruit more CD4^+^ T-cells and CD8^+^ T-cells compared to normal keratinocyte controls (P ≤0.01), suggesting that an immunoregulatory and effector T cell response is triggered by oncogenic v-ras^Ha^ expression. A significantly increased number of CD8^+^ T-cells was observed in v-ras^Ha^/ΔNp63α tumors (P ≤0.05), albeit with a high degree of variability. However, Regulatory T-cells (Tregs) are recruited at ~2-fold higher number to v-ras^Ha^ (P ≤0.05) and v-ras^Ha^/ΔNp63α (P ≤0.001) tumors compared to normal keratinocyte grafts or intact skin, suggesting that oncogenic v-ras^Ha^ can concurrently drive recruitment of Tregs implicated in immunosuppression into the TME. Further, the tumors arising from v-ras^Ha^/ΔNp63α-transduced keratinocytes also have significantly greater numbers of Ly6G^hi^Ly6C^int^ PMN-like myeloid cells compared to grafts from v-ras^Ha^ alone, control keratinocytes or intact skin, suggesting that the v-ras^Ha^/ΔNp63α had a significant impact on the concurrent recruitment of these potentially immunosuppressive neutrophilic cells. Lower numbers of Ly6G^lo^Ly6C^hi^ monocytic myeloid cells (~100s/10,000 cells) compared to Ly6G^hi^Ly6C^int^ PMN-like myeloid cells (300-1500s/10,000 cells) were observed across samples in both v-ras^Ha^ and v-ras^Ha^/ΔNp63α tumors similar to control normal cell grafts. The number of NK cells recruited were also similar across primary keratinocytes, v-ras^Ha^/Stuffer, and v-ras^Ha^/ΔNp63α. Consistent with their role in innate immunity, NK cells were recruited to the wounding of the graft procedure independent of the oncogenes expressed.

Multiplex immunofluorescence (multiplex IF) staining was used as an orthogonal method to visualize and determine the level of immune infiltrates in the tumors (**Figure 5B**). Consistent with the flow cytometry results (**Figure 5A**), v-ras^Ha^/ΔNp63α tumors have significantly increased numbers of Ly6G^hi^ neutrophilic myeloid cells (**Figure 5C**). Tumors stained for CD4^+^, CD8^+^, and FoxP3^+^ (Tregs) show no significant differences between the v-ras^Ha^ and v-ras^Ha^/ΔNp63α tumors (**Figure 5C**). Together, these data indicate that v-ras^Ha^/ΔNp63α-induced carcinomas recruit increased numbers of Ly6G^hi^ PMN-like cells in the TME. Together, the flow cytometry and IF data suggest that expression of ΔNp63 in SCC supports the induction of cells that bear PMN-MDSC markers as well as CD4^+^ and CD8^+^ T cell markers.

**Figure 6:**
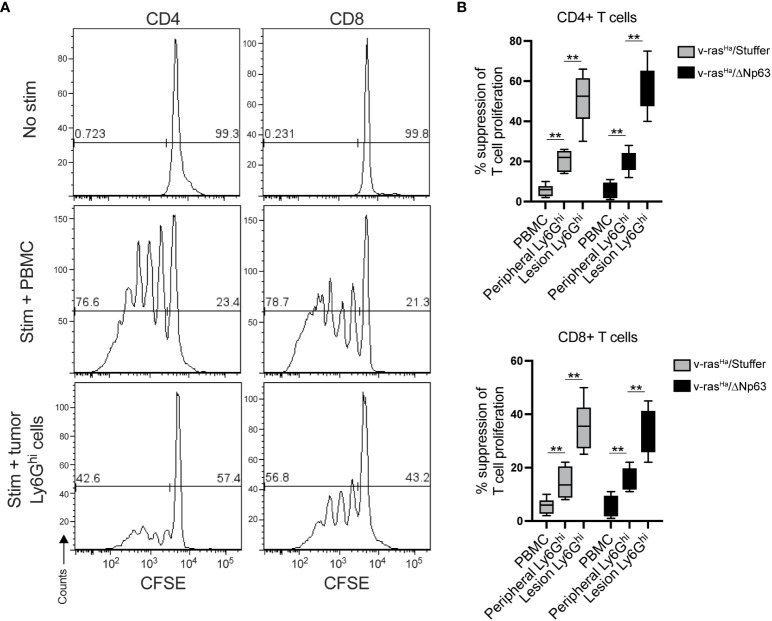
Lesion infiltrating Ly6G^hi^ cells are PMN-MDSCs. Ly6G^hi^ myeloid cells were isolated from the spleens or tumors of mice bearing lesions derived from primary keratinocytes encoding *v-ras^Ha^* alone (v-ras^Ha^/Stuffer) or in combination with *ΔNp63* (v-ras^Ha^/ΔNp63) and assessed for their ability to suppress the proliferation of CD3/28 stimulated wild-type CFSE-labelled CD4^+^ and CD8^+^ T cells in comparison to total splenocytes (PBMC). Proliferation was assessed by flow cytometric analysis. **(A)** Representative CFSE histograms of unstimulated T cells (top panels), T cells co-cultured with splenocytes (middle panels), or Ly6G^hi^ cell isolated from a v-ras^Ha^/ΔNp63 lesion. **(B)** Quantification of % suppression of T cell proliferation. Data pooled from three experimental replicates. **, *p* < 0.01. Stuffer = Empty Vector.

In mice, the phenotype of neutrophils is very similar to that of immunosuppressive neutrophilic myeloid derived suppressor cells (PMN-MDSCs), and the distinction between PMN-MDSCs and neutrophils requires functional assays (45). In order to distinguish PMN-MDSCs from neutrophils in this context, we determined whether neutrophilic populations isolated from tumors and spleen are capable of inhibiting T-cell proliferation. At the base line, cells without stimulation result in a single peak, indicating the absence of proliferation (**Figure 6A**, top panel). When stimulated with antibodies to CD3 and CD28, CD4^+^ and CD8^+^ T-cells proliferate in the presence of non-specific control PBMCs (from splenocytes), indicated by the progressive dilution of CSFE dye after a few days (**Figure 6A**, middle panel). However, in the presence of Ly6G^hi^ cells isolated from the tumors, the extent of proliferation was significantly inhibited upon stimulation (**Figure 6A**, bottom panel). Quantitation of the suppressive activity of tumor- and spleen- derived Ly6G^hi^ cells indicate that both populations inhibit proliferation but to a different degree (**Figure 6B**). The Ly6G^hi^ cells from the tumors inhibited T cell proliferation to a significantly greater degree than peripheral Ly6G^hi^ cells (**Figure 6B**). These data demonstrate that the neutrophilic cells that were recruited into v-ras^Ha^/Stuffer and v-ras^Ha^/ΔNp63α tumors are PMN-MDSCs.

## 4 Discussion

Among 33 cancer types analyzed by Pan TCGA, the *TP63* and *HRAS* genes are significantly overexpressed in human head and neck and lung SCCs compared to normal tissues (**Figures 1A, B**). Both genes are overexpressed more significantly in the advanced stage HNSCCs, in which expression levels of both genes rank the highest among the major cancer types analyzed, suggesting pathological significance. The elevated *TP63* gene expression resulting from 3q amplification and preferential expression of *ΔNp63* isoforms are consistent with previous reports (2, 5). *RAS* genes are frequently mutated across cancer types; however it has been reported that *HRAS* mutations are relatively low frequency overall, and associated primarily with squamous cancers. The *HRAS* mutation frequency varies depending on studies, with up to 20% reported in cutaneous SCCs, and up to 6% reported in HNSCCs (1, 3, 4, 6, 48–50). Based on our analysis of the TCGA data, the degree of overexpressed *HRAS* in HNSCCs is significant (**Figures 1A, B**). This is consistent with a previous finding that wild-type overexpressed *HRAS* is at a significantly higher frequency in human HNSCCs than that of the mutated HRAS (49). Interestingly, the prognosis of HNSCCs may be different based on the mutational status of *HRAS* (51). Further analyses will be needed to investigate the role of RAS mutational status/expression level in the immune TME and how it relates to the clinical outcome of SCCs. Nonetheless, this information emphasizes that the activation of the Ras pathway, whether achieved by the overexpression of wild-type gene or oncogenic gain-of-function mutation, plays an important role in the pathogenesis of SCCs. As suggested by analyses of single cell RNA-seq data from human HNSCCs, the contribution of RAS and ΔNp63 oncogenes are derived from epithelial cells and not from the stromal or immune cells within the tumors (**Figure 2**).

As reported here, in this mouse model, significantly elevated levels of immunosuppressive PMN-MDSCs are recruited along with CD8^+^ T cells into v-ras^Ha^/ΔNp63α-driven carcinomas compared to v-ras^Ha^-initiated tumors by 14 days. We established that cells from these tumor-bearing hosts expressing these PMN-MDSC markers functionally suppress T cells. These results are consistent with the secretion of chemokines transcriptionally inducible by ΔNp63 and implicated in the recruitment of PMN-MDSCs to the TME (21, 22). Notably, these findings are consistent with bioinformatic analyses from an earlier Pan-Squamous TCGA study showing that human SCC that express ΔNp63 are concurrently infiltrated with immune cells bearing CD4^+^ and CD8^+^ T cell markers along with cells expressing MDSC and immunosuppressive markers (2).

The contribution of MDSCs to tumor progression has been studied extensively (45). Although MDSCs are practically undetectable in healthy individuals, increasing numbers of circulating MDSCs correlate with stage, metastasis, tumor burden, and a worse prognosis in various cancers. MDSCs exert an immunosuppressive function locally within the TME as well as systemically throughout the host (45). Proposed mechanisms of this immune suppression by MDSCs include depletion of local nutrients required for T-cell function, such as L-arginine and tryptophan, by producing enzymes such as arginase-1 (ARG1), nitric oxide synthase (NOS2), and indole amine 2,3 dioxygenase (IDO) (41). In the current study, we demonstrate that Ly6G^hi^ neutrophilic populations isolated from v-ras^Ha^/ΔNp63α tumors are PMN-MDSCs (**Figure 6**). Our data indicate that PMN-MDSCs in both premalignant and malignant tumors were more immunosuppressive than peripheral PMN-MDSCs, suggesting that the TME significantly polarizes recruited neutrophilic cells toward a more immunosuppressive state, consistent with previously reported studies (reviewed in (41), **Figure 6**). Levels of monocytic myeloid cells, which may include monocytic MDSCs and macrophages, were consistently low in the tumor site. This may reflect differential chemokines required for trafficking of monocytic cells into tumors.

The accumulation of inflammatory cells including MDSCs in cancer is attributed to the production of cytokines such as GM-CSF, M-CSF, CCL2, CXCL2, and CXCL5 (41, 42). In the current study, our data demonstrate that ΔNp63α further promotes accumulation of tumor PMN-MDSCs within the tumor tissue, which correlated with the increased levels of chemokines *Cxcl1* and *Cxcl5*. This observation is in line with previous studies demonstrating the dependence of tumor growth on host immune cells (52, 53). Early work by Pekarek et al. demonstrated the role of granulocytes in rapid growth of tumor cells *in vivo* (52), and overexpression of CXCL1 (KC, Gro-α/Gro1) in the PAM 212 murine SCC cell line yields larger and more aggressive tumors upon subcutaneous transplantation (53). This is linked to enhanced inflammatory and angiogenic responses, dependent on infiltration of CXCR2 expressing granulocytes from the host (52, 53). Similarly, the role of ΔNp63 in the recruitment of tumor PMN-MDSCs has been demonstrated in a mouse tumor model of triple negative breast cancer (TNBC), a disease which shares common genetic and molecular features of squamous-like cancer subtype, including overexpression of ΔNp63 (5, 54). In the syngeneic mouse model of TNBC, a mammary epithelial cell line expressing oncogenic v-ras^Ha^ and ΔNp63-induced the recruitment of PMN-MDSCs to the primary tumor and metastatic sites. Chemokines, CCL22 and CXCL2 were identified as important effectors of MDSC recruitment into these ΔNp63 expressing TNBC tumors (54).

Our *in vitro* chemokine data indicated that v-ras^Ha^ alone induces a significant level of chemokines (CXCL2, CXCL5, CXCL7) that recruit inflammatory cells (**Supplementary Table S1**). This is consistent with previous observations that overexpression of v-ras^Ha^ in keratinocytes activates EGFR signaling leading to activation of IL-1α, NF-κB, and CXCR2 ligands, important mediators of tumorigenesis (13, 14). Interestingly, our data indicate that *in vitro*, ΔNp63α overexpression alone resulted in a minimal impact on chemokine production compared to v-ras^Ha^ or Stuffer control primary keratinocytes (**Supplementary Figure 3**). This pattern was confirmed by three independent methods: cytokine array, Bioplex (**Supplementary Figure 2C**), and the same custom RT^2^ profiler array used for *in vivo* chemokine detection (**Supplementary Figure 4**). These *in vitro* results may partly explain why the overexpression of ΔNp63α by itself is not sufficient to initiate tumors and that ΔNp63α requires cooperation with additional oncogene(s) (i.e., RAS) to promote malignant conversion (12, 15, 21, 22, 55). We have shown that ΔNp63 cooperates with NF-kB to promote cytokine gene expression (21), and Ras has been shown to be an inducer of NF-kB (56). It is possible that the expression levels of ΔNp63 in tumors is significantly higher relative to what occurs *in vitro*, which may be explained by the paracrine and autocrine signaling in the TME. In an autochthonous murine model of p63-induced SCC tumors, ΔNp63 expression was significantly higher in tumors compared to cultured cells (57). In addition, paracrine signaling between tumor cells and surrounding cells such as fibroblasts and macrophages, which can activate chemokines within the TME, may play a role in ΔNp63-dependent tumorigenesis. Such paracrine signaling between ΔNp63-overexpressing cells has been reported in other tumor models (54, 57). Likewise, the expression of ΔNp63 has been shown to be induced by TGF-β *via* Smad2 and IKKα in the A431 epidermoid carcinoma cell line (58). Preferential expression of ΔNp63 by hypomethylation of the *ΔNp63* transcriptional start site is also observed in SCCs (2), supporting that other factors (i.e. TGF-β, epigenetic regulation) may contribute to increased ΔNp63 expression independently of 3q amplification. This underscores the dynamic interplay between the ΔNp63 and the TME.

Taken together, our data suggest that ΔNp63α in cooperation with v-ras^Ha^ promotes an immunosuppressive TME through production of immune cell chemokines and recruitment of PMN-MDSCs and Tregs. Our previous studies have demonstrated cross-talk between v-ras^Ha^, ΔNp63α, and NF-κB signaling pathways implicated in squamous tumorigenesis (18, 21, 22) and highlight a potential role of NF-κB/c-Rel signaling together with ΔNp63α in the recruitment of PMN-MDSCs. Moreover, NF-κB, which has been shown to be essential in two-stage skin carcinogenesis (59), imparts survival of mutant Ras-expressing MEFs from macrophage-induced apoptosis and overcomes immune surveillance *via* regulation of gene expression that enriches the MDSC population, thereby facilitating a tumorigenic phenotype (60). PMN-MDSCs are increasingly recognized as an important target within the TME for their overarching role in cancer progression and that has been targeted in clinical trials in cancer patients (61). The data presented here enhance our understanding of the link between underlying genomic alterations commonly present within carcinomas and the development of an immunosuppressive TME. This engrafted keratinocyte model adapted to a syngeneic murine background may serve as a valuable tool in future interventional studies aimed at abrogating tumor immunosuppression.

## Data availability statement

The original contributions presented in the study are included in the article/**Supplementary Material**. Further inquiries can be directed to the corresponding author.

## Ethics statement

The animal study was reviewed and approved by Animal Care and Use Committee of the Center for Biologics Evaluation and Research of the Food and Drug Administration.

## Author contributions

NS, VG, RP and WW, optimized and performed the *in vivo* grafting studies. PC and BW performed the tumor processing and characterization of immune subsets, multiplex staining, analysis of chemokines from tumors. NS performed the *in vitro* studies with primary keratinocytes including analysis of chemokine secretion. CS performed the computational analyses of isoform and gene expression data from TCGA and scRNA-seq data. ZC contributed to the data analysis, graphic presentation, and interpretation of TCGA results across comparison of 33 cancer types. NS and PC wrote the first draft of the manuscript. All authors contributed to the article and approved the submitted version. WW, CW, and CA, oversaw the study.

## Supplementary material

Supplementary Figure 1 Grafting of keratinocytes overexpressing ΔNp63 alone does not give rise to tumors. Primary control keratinocytes transduced with viruses encoding *ΔNp63* alone or in combination with *v-ras^Ha^* were combined with cultured primary dermal cells and grafted onto the dorsal surfaces of wildtype BALB/c mice. The pictures were taken 4 weeks post grafting. Grafts of v-ras^Ha^/ΔNp63 expressing cultures are included as positive controls. These results are consistent with results previously described in immune deficient mice (12).

Supplementary Figure 2 H&Es of lesions. Representative photomicrographs (10X lens, Zeiss Axio Vert) H&E sections of 2 and 4-week-old lesions derived from grafted primary keratinocytes that had been transduced with virus encoding *v-ras^Ha^* alone (v-ras^Ha^/Stuffer) or in combination with *ΔNp63* (v-ras^Ha^/ΔNp63). Stuffer = Empty control vector. Each image represents an independent tumor from a different animal.

Supplementary Figure 3
*In Vitro* Chemokine and Cytokine Assays. Cultured primary keratinocytes overexpressing Ras release chemokines involved in recruiting infiltrating immune cells. *in vitro* Supernatants from primary keratinocytes following transduction with retrovirus encoding v-ras^Ha^ (v-ras^Ha^/Stuffer), empty vector (Stuffer), ΔNp63α (ΔNp63), or combination of v-ras^Ha^ and ΔNp63α (v-Ras^Ha^/ΔNp63) were collected for chemokine and cytokine detection. **(A, B)** Three days following the final transduction as described in Methods, the cell culture medium was replaced with fresh medium and 24 hours later the supernatant was collected and used immediately to probe for chemokines and cytokines (as indicated in the table, upper panel). Culture supernatants were overlaid on array membranes (bottom panels) C3 **(A)** and C4 **(B)** and incubated overnight at 4˚C. The chemiluminescence signal was detected using Amersham ImageQuant LAS 4000. Results shown are representative of 3 independent experiments. The dot blot results were qualitatively examined across triplicate results, and the chemokines significantly up-regulated by v-ras^Ha^ (and v-ras^Ha^/ΔNp63) are marked by red boxes, and modestly upregulated chemokines are marked by green boxes. Modest down-regulation by *v-ras^Ha^* was seen in some chemokines, indicated by blue boxes. A list of the modulated chemokines is provided in **Supplementary Table S1**. **(C)** ProcartaPlex *in vitro* quantitative immunoassay was performed according to manufacturer’s instructions to measure the relative levels of chemokines present in the supernatants. The supernatants were collected 4- and 14-days post-final transduction. Each sample was tested in duplicate and averaged. Results presented represent the mean and standard deviation from 3 independent experiments. The mean of the results were compared to Stuffer and analyzed using 2way ANOVA using GraphPad Prism 9.4.0 (*, *p* < 0.05; **, *p* < 0.01; ***, *p* < 0.001).

Supplementary Figure 4
*In vitro* chemokine expression analysis by RT^2^ profiler PCR analysis. Custom RT^2^ qPCR profiler (also used for the detection of chemokine and receptor expression in tumors presented in **Figure 4**) was used to assess the chemokine gene expression in the RNA extracts of cultured BALB/c newborn primary keratinocytes transduced with retrovirus encoding v-ras^Ha^ (v-ras^Ha^) followed by empty vector (Stuffer) or ΔNp63α (ΔNp63) as described in Methods. Four days following the first transduction (v-ras^Ha^), the RNA was collected and RT^2^ qPCR was performed according to the manufacturer’s protocol. Values were normalized to GAPDH as a housekeeping gene. The results were analyzed using the RT^2^ profiler PCR data analysis online tool available on Qiagen’s website (https://geneglobe.qiagen.com/us/analyze). Each sample was tested in duplicate and averaged, and the fold change compared to Stuffer control was plotted using GraphPad Prism 9.2.0. The results presented are from three independent experiments. The mean of the results were compared to Stuffer and analyzed using 2-way ANOVA using GraphPad Prism 9.4.0 (*, *p* < 0.05; **, *p* < 0.01; ***, *p* < 0.001).
