# Supplementary figures and images for: Oncogenic Ras and ΔNp63α cooperate to recruit immunosuppressive polymorphonuclear myeloid-derived suppressor cells in a mouse model of squamous cancer pathogenesis
Source: Front Immunol. 2023 Aug 10;14:1200970. doi: 10.3389/fimmu.2023.1200970 (PMC10449460; doi:10.3389/fimmu.2023.1200970)

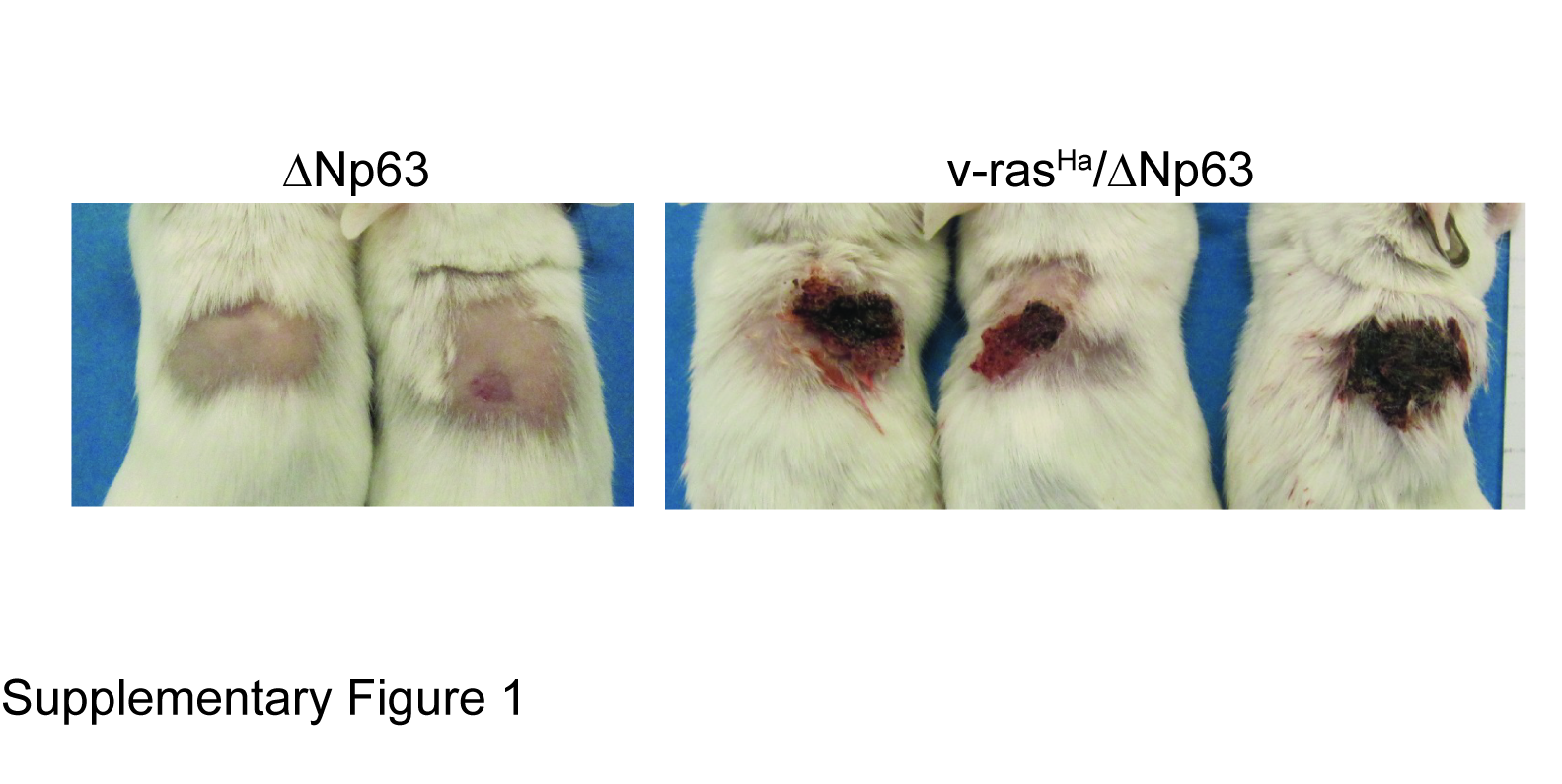

Supplement: Supplementary Figure 1 — Grafting of keratinocytes overexpressing ΔNp63 alone does not give rise to tumors. Primary control keratinocytes transduced with viruses encoding ΔNp63 alone or in combination with v-rasHa were combined with cultured primary dermal cells and grafted onto the dorsal surfaces of wildtype BALB/c mice. The pictures were taken 4 weeks post grafting. Grafts of v-rasHa/ΔNp63 expressing cultures are included as positive controls. These results are consistent with results previously described in immune deficient mice (12). [file Image_1.tif]

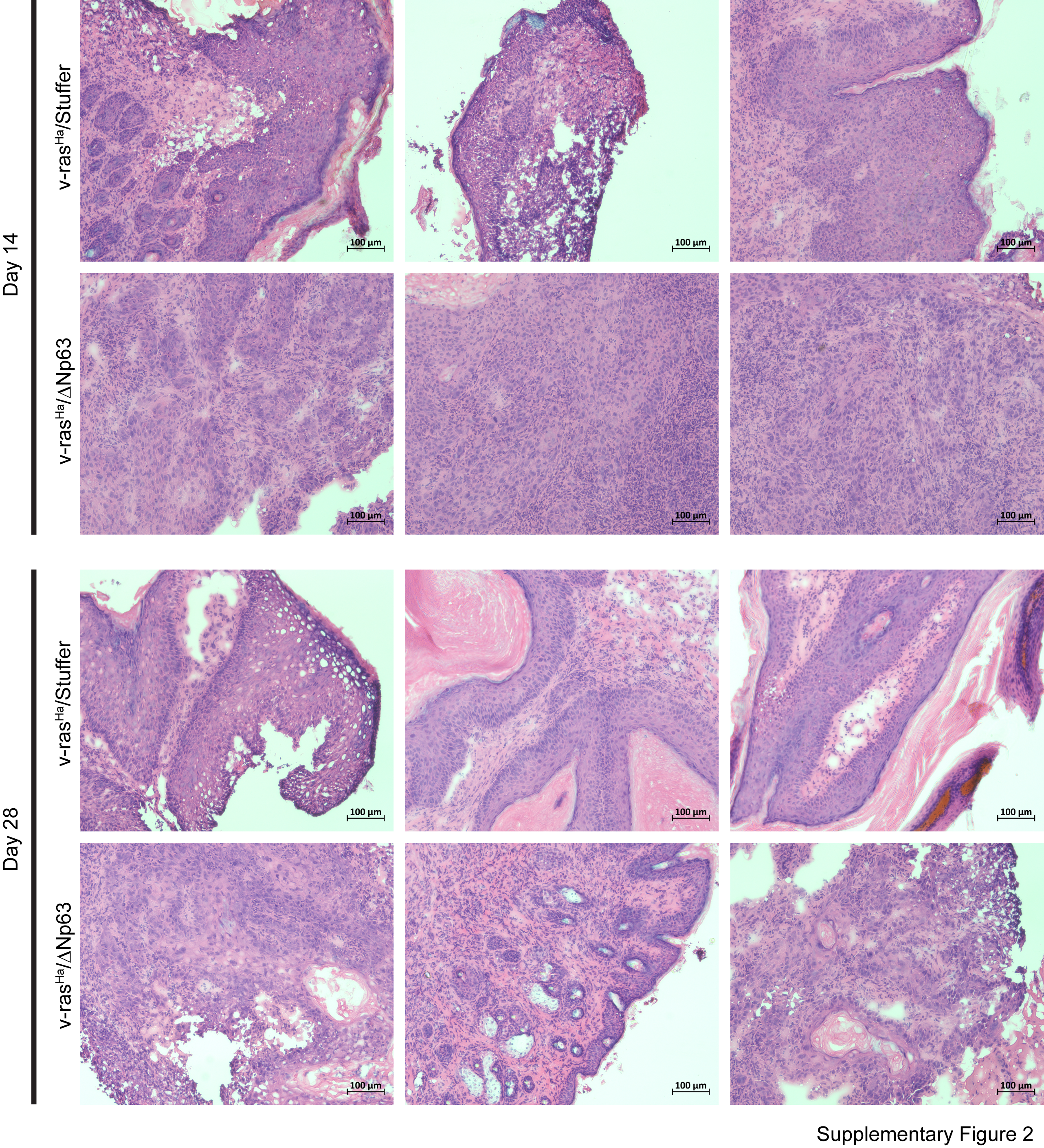

Supplement: Supplementary Figure 2 — H&Es of lesions. Representative photomicrographs (10X lens, Zeiss Axio Vert) H&E sections of 2 and 4-week-old lesions derived from grafted primary keratinocytes that had been transduced with virus encoding v-rasHa alone (v-rasHa/Stuffer) or in combination with ΔNp63 (v-rasHa/ΔNp63). Stuffer = Empty control vector. Each image represents an independent tumor from a different animal. [file Image_2.tif]

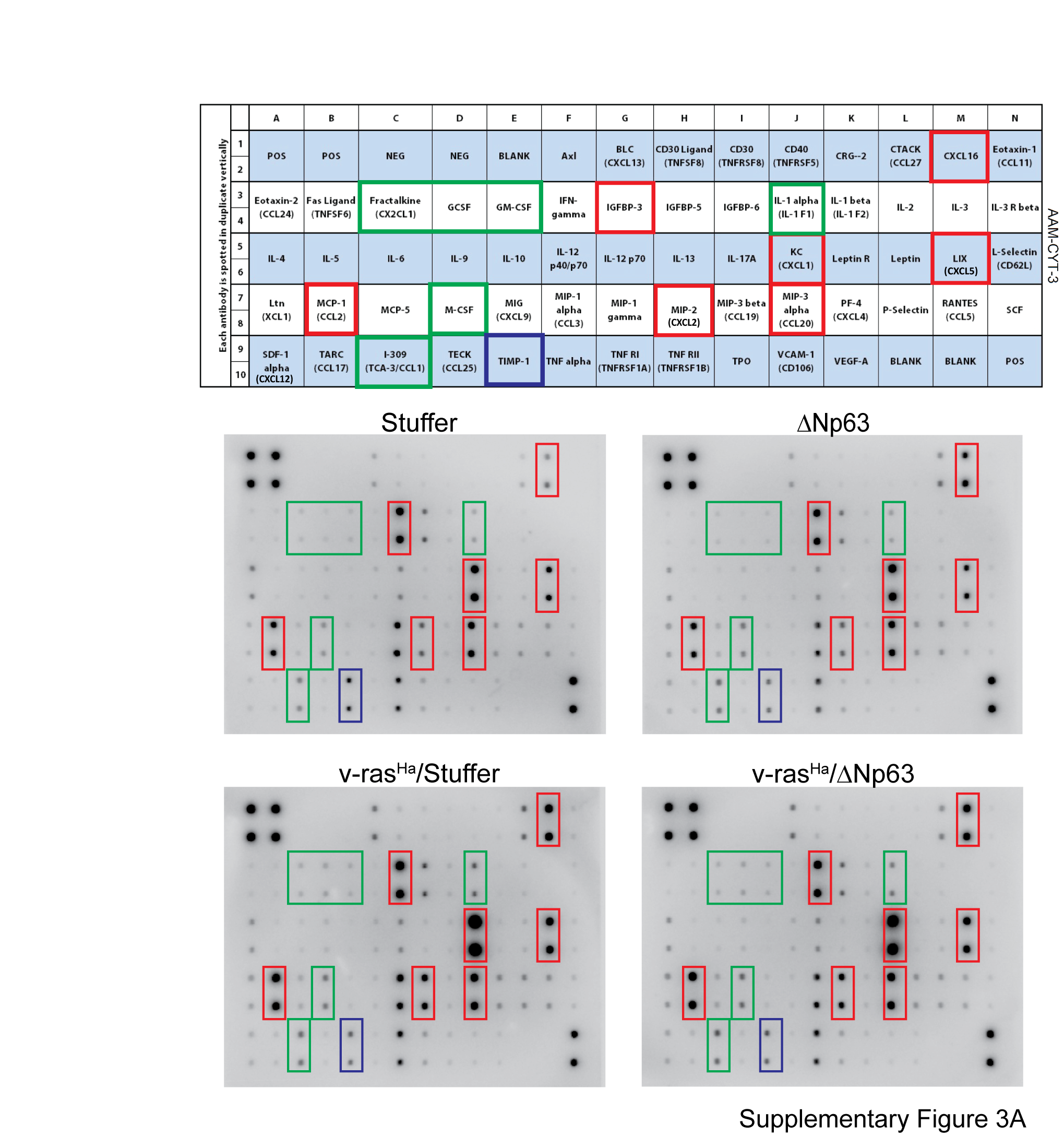

Supplement: Supplementary Figure 3 — In Vitro Chemokine and Cytokine Assays. Cultured primary keratinocytes overexpressing Ras release chemokines involved in recruiting infiltrating immune cells. in vitro Supernatants from primary keratinocytes following transduction with retrovirus encoding v-rasHa (v-rasHa/Stuffer), empty vector (Stuffer), ΔNp63α (ΔNp63), or combination of v-rasHa and ΔNp63α (v-RasHa/ΔNp63) were collected for chemokine and cytokine detection. (A, B) Three days following the final transduction as described in Methods, the cell culture medium was replaced with fresh medium and 24 hours later the supernatant was collected and used immediately to probe for chemokines and cytokines (as indicated in the table, upper panel). Culture supernatants were overlaid on array membranes (bottom panels) C3 (A) and C4 (B) and incubated overnight at 4˚C. The chemiluminescence signal was detected using Amersham ImageQuant LAS 4000. Results shown are representative of 3 independent experiments. The dot blot results were qualitatively examined across triplicate results, and the chemokines significantly up-regulated by v-rasHa (and v-rasHa/ΔNp63) are marked by red boxes, and modestly upregulated chemokines are marked by green boxes. Modest down-regulation by v-rasHa was seen in some chemokines, indicated by blue boxes. A list of the modulated chemokines is provided in Supplementary Table S1 . (C) ProcartaPlex in vitro quantitative immunoassay was performed according to manufacturer’s instructions to measure the relative levels of chemokines present in the supernatants. The supernatants were collected 4- and 14-days post-final transduction. Each sample was tested in duplicate and averaged. Results presented represent the mean and standard deviation from 3 independent experiments. The mean of the results were compared to Stuffer and analyzed using 2way ANOVA using GraphPad Prism 9.4.0 (*, p < 0.05; **, p < 0.01; ***, p < 0.001). [file Image_3.tif]

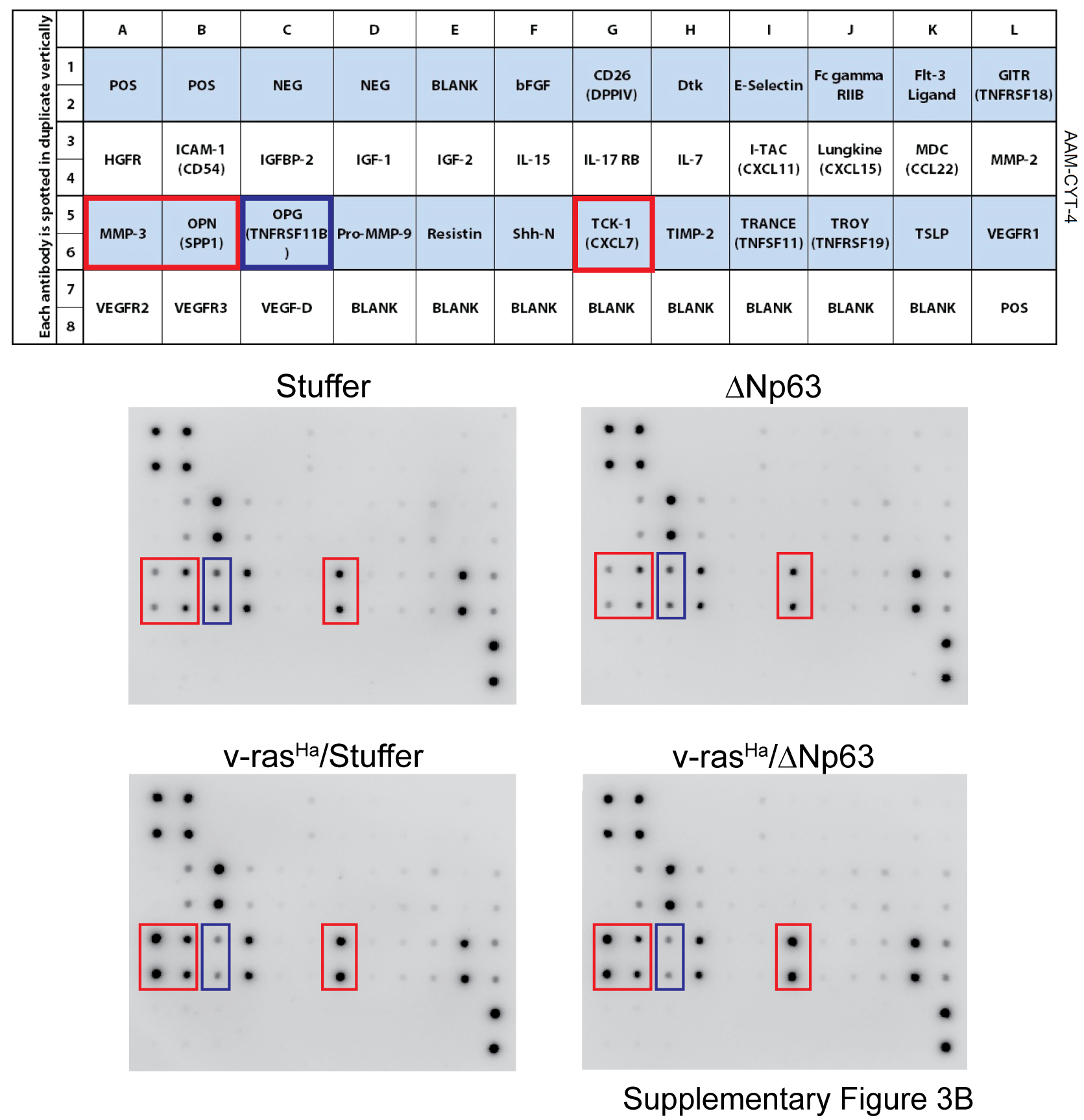

Supplement: Supplementary Figure 4 — In vitro chemokine expression analysis by RT2 profiler PCR analysis. Custom RT2 qPCR profiler (also used for the detection of chemokine and receptor expression in tumors presented in Figure 4 ) was used to assess the chemokine gene expression in the RNA extracts of cultured BALB/c newborn primary keratinocytes transduced with retrovirus encoding v-rasHa (v-rasHa) followed by empty vector (Stuffer) or ΔNp63α (ΔNp63) as described in Methods. Four days following the first transduction (v-rasHa), the RNA was collected and RT2 qPCR was performed according to the manufacturer’s protocol. Values were normalized to GAPDH as a housekeeping gene. The results were analyzed using the RT2 profiler PCR data analysis online tool available on Qiagen’s website (https://geneglobe.qiagen.com/us/analyze). Each sample was tested in duplicate and averaged, and the fold change compared to Stuffer control was plotted using GraphPad Prism 9.2.0. The results presented are from three independent experiments. The mean of the results were compared to Stuffer and analyzed using 2-way ANOVA using GraphPad Prism 9.4.0 (*, p < 0.05; **, p < 0.01; ***, p < 0.001). [file Image_4.tif]

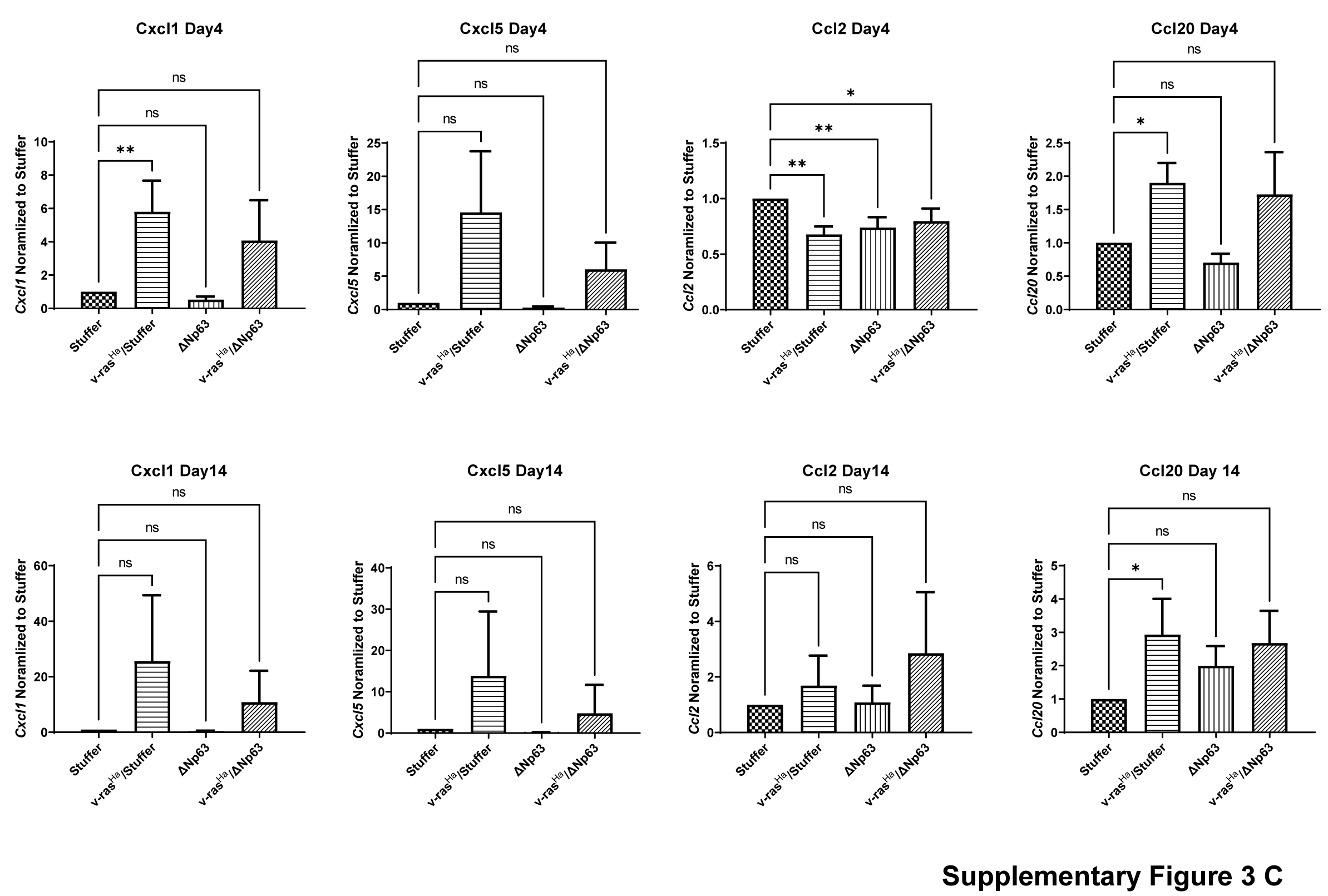

Supplement: Supplementary file 5 [file Image_5.tif]

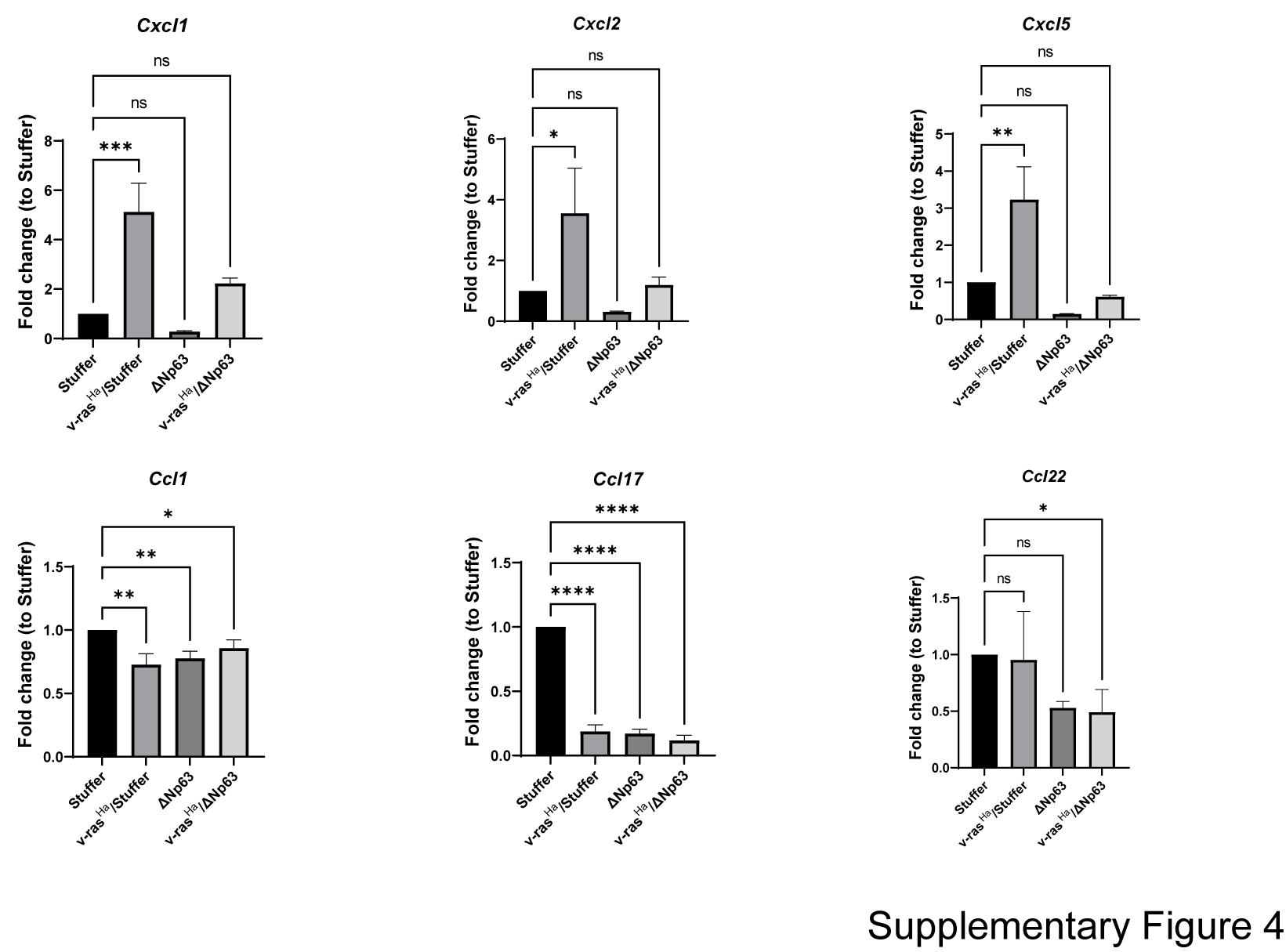

Supplement: Supplementary file 6 [file Image_6.tif]
