# Supplementary material for: Oncogenic Ras and ΔNp63α cooperate to recruit immunosuppressive polymorphonuclear myeloid-derived suppressor cells in a mouse model of squamous cancer pathogenesis
Source: Front Immunol. 2023 Aug 10;14:1200970. doi: 10.3389/fimmu.2023.1200970 (PMC10449460; doi:10.3389/fimmu.2023.1200970)
Supplement: Supplementary file 7 [file Table_1.docx]

**Supplementary Table S1.** List of highlighted secreted factors, chemokine and cytokines.

| Up-regulation by v-ras^Ha^ /Stuffer and v-ras^Ha^ /ΔNp63 (Red) | Modest up-regulation by v-ras^Ha^ /Stuffer and v-ras^Ha^ /ΔNp63 (Green) | Modest down-regulation by v-ras^Ha^ /Stuffer and v-ras^Ha^ /ΔNp63 (Blue) |
| --- | --- | --- |
| CXCL1 | Fractalkine (CX3CL1) | OPG (TNFRSF11B) |
| CXCL2 | GCSF | TIMP-1 |
| CXCL5 | GM-CSF |  |
| CXCL7 | IL-1F1 |  |
| CXCL16 | M-CSF |  |
| CCL2 | CCL1 |  |
| CCL20 |  |  |
| IGFBP-3 |  |  |
| MMP-3 |  |  |
| OPN (SPP1) |  |  |
